# Potential distribution patterns and species richness of avifauna in rapidly urbanizing East China

**DOI:** 10.1002/ece3.11515

**Published:** 2024-06-18

**Authors:** Wan Chen, Xuan Wang, Yuanyuan Cai, Xinglong Huang, Peng Li, Wei Liu, Qing Chang, Chaochao Hu

**Affiliations:** ^1^ College of Environment and Ecology Jiangsu Open University (The City Vocational College of Jiangsu) Nanjing Jiangsu China; ^2^ Jiangsu Academy of Forestry Nanjing Jiangsu China; ^3^ College of Life Sciences Nanjing Normal University Nanjing Jiangsu China; ^4^ Yangzhou Urban Forest Ecosystem National Research Station Yangzhou Jiangsu China; ^5^ Shanghai International Airport Co., Ltd. Pudong International Airport Shanghai China; ^6^ Nanjing Institute of Environmental Sciences, Ministry of Environmental Protection Nanjing Jiangsu China; ^7^ Analytical and Testing Center Nanjing Normal University Nanjing Jiangsu China

**Keywords:** bird diversity, protection gap, species distribution, species richness

## Abstract

In recent years, increased species extinction and habitat loss have significantly reduced biodiversity, posing a serious threat to both nature and human survival. Environmental factors strongly influence bird distribution and diversity. The potential distribution patterns and species richness offer a conservation modeling framework for policymakers to assess the effectiveness of natural protected areas (PAs) and optimize their existing ones. Very few such studies have been published that cover a large and complete taxonomic group with fine resolution at regional scale. Here, using birds as a study group, the maximum entropy model (MaxEnt) was used to analyze the pattern of bird species richness in Jiangsu Province. Using an unparalleled amount of occurrence data, we created species distribution models (SDMs) for 312 bird species to explore emerging diversity patterns at a resolution of 1 km^2^. The gradient of species richness is steep, decreasing sharply away from water bodies, particularly in the northern part of Jiangsu Province. The migratory status and feeding habits of birds also significantly influence the spatial distribution of avian species richness. This study reveals that the regions with high potential bird species richness are primarily distributed in three areas: the eastern coastal region, the surrounding area of the lower reaches of the Yangtze River, and the surrounding area of Taihu Lake. Compared with species richness hotspots and existing PAs, we found that the majority of hotspots are well‐protected. However, only a small portion of the regions, such as coastal areas of Sheyang County in Yancheng City, as well as some regions along the Yangtze River in Nanjing and Zhenjiang, currently have relatively weak protection. Using stacked SDMs, our study reveals effective insights into diversity patterns, directly informing conservation policies and contributing to macroecological research advancements.

## Graphical abstract

This study used species distribution models for 312 bird species to find patterns of bird species richness and identify hotspots in Jiangsu Province. The results showed that most hotspots are well protected, but some areas, like parts of Sheyang County and regions along the Yangtze River, have weak protection measures.

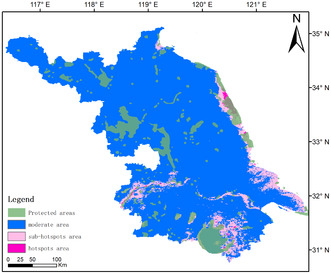

## 1 INTRODUCTION

Biodiversity plays a crucial role in maintaining ecological balance, and the conservation of biodiversity is one of the key factors in protecting the ecological environment (Mi et al., 2021). In recent years, global biodiversity loss has become severe due to factors like environmental pollution and climate change (Adawaren et al., 2020). With the increasing environmental challenges brought about by factors such as urban expansion and climate change, biodiversity is facing severe challenges (Li et al., 2023; Piaggio et al., 2017). Biodiversity loss threatens ecosystem productivity and services worldwide, affecting human well‐being mainly through affecting the functioning of natural ecosystems (Xu et al., 2017). In the face of rapid biodiversity loss, attention has been increasingly focused on the application of maps toward the challenges of protecting biodiversity (Cardinale et al., 2012; Dri et al., 2021). Efficient conservation and monitoring initiatives rely on understanding species distributions and associated diversity patterns scientifically (Herkt et al., 2016; Hu et al., 2017). Analyzing biodiversity distribution and identifying hotspots are valuable for conservation (Huang et al., 2020; Yang et al., 2021), offering crucial insights for conservation and management purposes (Zhang et al., 2019). However, biodiversity maps can lead, or have led us into, errors since they are too often not questioned by ecologists, who perceive them as an objective and legitimate representation of the natural world (Malavasi, 2020).

Species distribution models (SDMs) simulate species habitat suitability on different spatial scales by correlating species' presence with environmental characteristics (Dias et al., 2023; Warren, 2012). Modeling species distribution has become a key tool in conservation, ecology, biogeography, evolution, invasive species control, and wildlife management studies (Huang et al., 2020; Li et al., 2020). Others consider that SDMs, using data collected from museums, herbariums, or field surveys, are vital for the identification of priorities for biodiversity conservation, targeting endemic, or endangered species (Pascual et al., 2011). However, insufficient data are available for the majority of threatened species, hampering conservation management measures to evaluate the protected areas (PAs) network (Grand et al., 2007). Conservation gap analysis assesses the adequacy of PAs in aligning with conservation priorities. Such analysis involves modeling conservation priorities, and then comparing them against those in PAs, which play an important role in protecting biodiversity and securing ecosystem services (Huang et al., 2020).

The maximum entropy (MaxEnt) model has been widely used to model potential species distribution, using species sites and environmental variables as inputs (Phillips et al., 2006). MaxEnt is among the most used SDM techniques, principally because it can be used with either presence‐only data or presence–absence data (Venne & Currie, 2021). However, in most cases, the real state of land cover is ignored when using the MaxEnt model, which may lead to exaggerated outputs (Han et al., 2019). Regarding the research on the potential distribution of species, most studies are applied to the comparison of one or several species (Anibaba et al., 2022; Sierra‐Morales et al., 2021). The accuracy of SDMs is greatly influenced by the quality and quantity of input data and the choice of SDM algorithm and model (Moonlight et al., 2020; Scherrer et al., 2021). However, studying a specific major biological group within a particular range using large‐scale species distribution data is relatively limited (Carroll et al., 2023; Hu et al., 2017).

Based on different scales, current research can be broadly categorized into three main groups. On a broader scale, such as national or international, the identification of factors influencing distribution patterns appears plausible due to the extensive range of climate variations (He et al., 2017; Yao et al., 2022). Due to the inherent difficulty in acquiring the necessary data, many studies resort to analyzing information from diverse indirect sources, posing challenges to ensuring the accuracy of their findings. Conversely, on a smaller scale, such as the county or city level, rigorous research has been undertaken through field surveys (Galitsky & Lawler, 2015). Nevertheless, the use of diverse methodologies in these studies poses a challenge when comparing the distribution patterns of communities across different regions. In contrast, at a moderate scale (e.g., provincial or regional), there is a comparatively limited body of research addressing the structure of avian communities (Liang et al., 2019). Investigations at this scale have the potential to elucidate spatial variations in avian community structures more effectively.

Detailed knowledge of the geographical distributions of species is crucial for developing precise biogeographical interpretations and conservation strategies, especially in poorly sampled countries or regions (Serrano et al., 2023). The need for accurate occurrence records and the lack of detailed data on species distributions is the “Wallacean shortfall” (Oliveira et al., 2016). The limited distribution data are a challenge, leading to strong limitations in conservation efforts and spatial planning (Nemésio et al., 2024). SDMs play a crucial role in addressing the Wallacean shortfall by providing unbiased and easily interpretable estimates of species distribution information (Moonlight et al., 2020). They improve the accuracy and range of species distributions by incorporating ecological data such as climate and functional traits, along with fine‐scale aggregated data like specimen records, surveys, and experiments (López‐Aguilar et al., 2024; Silva et al., 2020). SDMs bridge the gap between fine‐scale precision and global representativeness, thus aiding in predicting and conserving biodiversity (Alvarez et al., 2021).

Here, we focused on birds diversity because they are well‐known and highly diverse in Jiangsu Province as well as having high levels of shorebird species exceeding 1% of the East Asian–Australasian Flyway (EAAF) population (Sun et al., 2021). Birds are easy to see, well‐studied, and important for ecosystems, which are widely recognized as key species for identifying and protecting important conservation areas (Hu et al., 2017; Lei et al., 2006). Currently, research on avian community ecology encompasses various topics, including field surveys, spatial distribution, community structure, the impact of environmental factors on community structure, and community assembly models (Adorno et al., 2021; Rushing et al., 2020; Wang et al., 2020; Yang et al., 2020). Most of these studies focus on species diversity and spatial variation (Hu et al., 2017; Huang et al., 2020). This lack of attention, combined with fast population growth and frequently high biodiversity in various regions, poses significant challenges (Chan et al., 2019; Jackson et al., 2019). Compared with less mobile species, the currently realized distributions of birds resemble closely their potential distributions (Carroll et al., 2023; Hu et al., 2017). Despite the province's size, studies on species distribution patterns and related conservation strategies in Jiangsu Province are lacking (Li & Zhang, 2021).

Studying bird diversity distribution patterns and identifying hotspots are crucial for biodiversity conservation efforts in Jiangsu Province, China. Therefore, this study primarily employs the combination of the MaxEnt model and GIS technology to explore the bird diversity hotspots in Jiangsu Province. Additionally, by integrating the ecological protected areas, the study aims to analyze the gaps in bird conservation, with the goal of providing a scientific basis for the protection and planning of bird species and protected areas in Jiangsu Province. The specific objectives of this study were to: (1) generate the potential spatial distribution of species richness for bird species in Jiangsu Province, (2) investigate the primary limiting variables influencing the habitat of bird species, and (3) map biodiversity hotspots while assessing conservation gaps in existing protected areas.

## 2 MATERIALS AND METHODS

### 2.1 Study area

This study was conducted in the Jiangsu Province, China, ranging from 116°18′ to 121°57′ E and 30°45′ to 35°20′ N. Since the start of economic reform in 1979, Jiangsu Province has become one of the fastest‐growing areas in China, and its GDP (Gross Domestic Products) reached RMB 12,822.21 billion in 2023, ranking the second in China. Jiangsu Province is situated in the plain of the middle and lower reaches of the Yangtze River, and it covers the coastal area along the Yangtze River, featuring many lakes, flat terrain, and a landscape marked by plains, water bodies, and low hills. Earlier research has shown that the size of the study area (area that is accessible) significantly influences the calibration of SDMs (Barve et al., 2011). It borders the Yellow Sea to the east and spans the two major river systems of the Yangtze and the Huaihe. Climatically, Jiangsu Province is situated in the transitional zone between the subtropical and warm temperate zones, featuring a mild climate, distinct seasons, and notable monsoons. The annual average temperature ranges from 13.6 to 16.1°C, with an annual precipitation of 704 to 1250 mm. The diverse natural geography and favorable climate provide a natural habitat for various wildlife. The scope of this study covers the entire Jiangsu Province, which has been converted into a grid system of 100 × 100 m using ArcGIS. All analyses will be conducted within this grid framework.

### 2.2 Species occurrence records

We compiled bird distribution data from long‐term field surveys, published papers, the China Bird Recording Center [CBRC; http://www.birdreport.cn/ (accessed on January 13, 2024)], and Global Biodiversity Information Facility (GBIF) database [http://www.gbif.org/ (accessed on January 13, 2024)] in Jiangsu Province, China. To evaluate the reliability of this data, we initially conducted checks for accuracy, consistency, and completeness to ensure its reliability. We also engaged professional taxonomists who are knowledgeable about the bird species in Jiangsu Province to contribute to the data quality assessment process. Subsequently, we eliminated occurrence records lacking precise location information. In cases where the exact geocoordinates were absent, we used Google Earth (http://ditu.google.cn/) to determine the latitude and longitude based on the provided geographical descriptions. We standardized the families, species names, and higher taxonomic nomenclature according to field guides for bird identification (Zheng, 2017). For each species, we also recorded the threat status based on the International Union for Conservation of Nature categories (IUCN, 2023).

In total, 125,646 occurrence records with 421 species were collected, of which many were duplicate records (GBIF.org, 2022). For this study, bird species with precise latitude and longitude coordinates recorded between 2013 and 2023 were selected. Species with 15 or more distribution data points were chosen for SDMs analysis (Prieto‐Torres et al., 2021). Duplicate and invalid records were manually removed; records with obvious misdescriptions of areas and inaccurate descriptions were discarded; and some records without clear geographical distribution were identified and confirmed using Google Earth and GPSspg [http://www.gpsspg.com/maps.htm/ (accessed on January 13, 2024)] for latitude and longitude. To minimize the sampling bias effect in our dataset (Boria et al., 2014), only one distribution point was randomly retained in a 2.5 arc minute grid cell. After removing points with unclear and incorrect information, we used the spThin package in R 4.0.1 for spatial rarefaction to examine the records for spatial autocorrelation, which helped to minimize spatial biases and ensure occurrence independence (Aiello‐Lammens et al., 2015). Finally, 312 bird species with 62,393 occurrence records were used for model calibration (Tables S1 and S2). In accordance with the requirements of MaxEnt software, the distribution records of the species were organized into “.csv” format files, which sorted by species name, longitude, and latitude of the distribution points.

### 2.3 Environmental predictors

Environmental variables are key factors affecting species distribution. Bioclimate data have been reported to be the most typically important variables for modeling potential species distribution (Bradie & Leung, 2017; Prieto‐Torres et al., 2021). In addition, altitude and land cover is an important environmental factor affecting the distribution of the birds (Venne & Currie, 2021). Thus, 19 bioclimatic factors, elevation, and land cover were identified as the key variables, which define the potential distribution niches for the birds and were used to establish the model. We obtained 19 bioclimatic and elevation variables at a resolution of 30 s (approximately 1 km^2^) using WorldClim (version 2.1; available at https://www.worldclim.org), which represented the average temperature and precipitation data from 1970 to 2000 (Fick & Hijmans, 2017). Problems such as multicollinearity among bioclimatic variables may lead to model overfitting, thus affecting the accuracy of the prediction results (Dai et al., 2018). To avoid the introducing redundant information, Pearson correlations were used to examine multicollinearity. In the pairwise comparison of the 19 bioclimatic variables, when the absolute value of the correlation coefficient of a certain climatic variable pair was greater than 0.7, it was regarded as a higher contribution, and the corresponding bioclimatic variable was eliminated (Warren et al., 2010). Based on the results of pairwise Pearson correlation analysis, we chose five climatic variables annual mean temperature (bio1), mean diurnal range/ temperature annual range (bio2); mean temperature of driest quarter (bio9); annual precipitation (bio12); and precipitation of wettest month (bio13) without collinearity to predict the SDM (Figure S1). The land cover (LC) data were collected from the Socioeconomic Data and Applications Center (https://sedac.ciesin.columbia.edu/) (Appendix Table S1). Finally, seven variables were selected to calibrate our models.

### 2.4 Species distribution modeling

To characterize the potential distribution based on ecological niche modeling, we used the maximum entropy model (MaxEnt 3.3) to create SDMs for 312 bird species with 62,393 occurrence records (Phillips et al., 2006). The maximum entropy model is widely used for predicting potential distribution and evaluating suitable habitats of target species. When the entropy value is maximum, the redundant information will be eliminated and the probability value of species existence will be generated, usually between 0 and 1. The two most important parameters of the model are the Feature combination (FC) that contains Linear (L), Quadratic (Q), Product (P), Threshold (T), and Hinge (H) and the Regularization Multiplier (RM). R package ENMeval was used to carry out the prediction operation of different parameter settings and generated a series of significant models with omission rates lower than 0.05. According to the Akaike information criterion (AICc), the FC and RM corresponding to the minimum value are to be used for the model. A 10‐fold cross‐validation approach was performed to ensure the predictive accuracy of models. The algorithm runs either 1000 iterations of these processes or continues until convergence (threshold 0.00001). The default values for the other parameters were used to build the habitat suitability model for bird species in Jiangsu Province.

The combination of the area under curve (AUC value) of receiver operating characteristic and the true skill statistic (TSS value) was used to assess the model performance (Allouche et al., 2006; Fielding & Bell, 1997). AUC varies between 0 and + 1: an AUC score between 1.0 and 0.9 = excellent, between 0.9 and 0.8 = good, between 0.8 and 0.7 = fair, between 0.7 and 0.6 = poor, and between 0.6 and 0.5 = fail (Swets, 1988). The TSS value is threshold dependent and calculated as: TSS = Sensitivity + Specificity – 1. TSS scores vary between −1 and +1, with a score close to 1 indicating an almost perfect model, while close to zero or less than zero indicates a model no better than random (Allouche et al., 2006). The average AUC and TSS across the 15 replicates of each algorithm across all species were used to assess model performance. The outputs were transformed into raster format using the ArcMap tool in ArcGIS software for further analysis.

### 2.5 Post‐processing

Based on the MaxEnt model predictions, the potential distribution maps of all bird species were overlaid using the raster calculator tool in ArcGIS to obtain the spatial distribution pattern of bird species richness in Jiangsu Province. To obtain species richness values per grid cell, we converted each species' continuous logistic MaxEnt output into a binary map (suitable/unsuitable) by Reclassify function in ArcGIS. Then, we calculated species richness by summing 312 binary SDM. Considering that the migratory status and feeding habits of birds may impact their potential distribution, we categorized the birds involved in the modeling into resident and migratory, as well as into omnivorous, herbivorous, and carnivorous feeding birds (Wilman et al., 2014). Additionally, studying the status of protected animals and threatened species is crucial for bird conservation. Therefore, we conducted analyses based on the conservation levels from the International Union for Conservation of Nature (IUCN) as well as the Chinese Class I and Class II protected bird species. We applied a natural break classification method depending on the species richness was applied in ArcGIS to classify areas into three levels: moderate areas (1–99), sub‐hotspots (100–149), and hotspots (150–182).

## 3 RESULTS

### 3.1 The composition of the bird community

Through long‐term field observations and downloading data from GBIF, this study found a total of 421 bird species over the past decade in Jiangsu Province, China. In consideration of the requirements for species distribution model analysis, this study ultimately selected 312 species for predicting the potential distribution zones, which belong to 18 orders and 59 families. The Passeriformes order includes 133 bird species from 31 families, representing 42.63% of all species. The next is the order Charadriiformes, with 62 species in 7 families, representing 19.87% of the total species. In terms of the composition of bird residency types, there are 82 resident species, accounting for 26.28% of the total species, and 230 migratory species, constituting 73.72% of the total species (Appendix Table S2).

The bird species used for modeling include 82 resident species, 230 migratory species, 196 carnivorous birds, 105 omnivorous birds, and 11 herbivorous birds. There are 2 birds classified as CR (Critically Endangered), 6 as EN (Endangered), 17 as NT (Near Threatened), and 17 as VU (Vulnerable) in terms of conservation status. Additionally, there are 10 species classified as first‐class national protection species in China in the List of National Key Protected Wild Animals revised version in 2021, representing the current important and endangered wild animals in the country; and 52 as second‐class national protection species in China.

### 3.2 Distribution models

**FIGURE 1:**
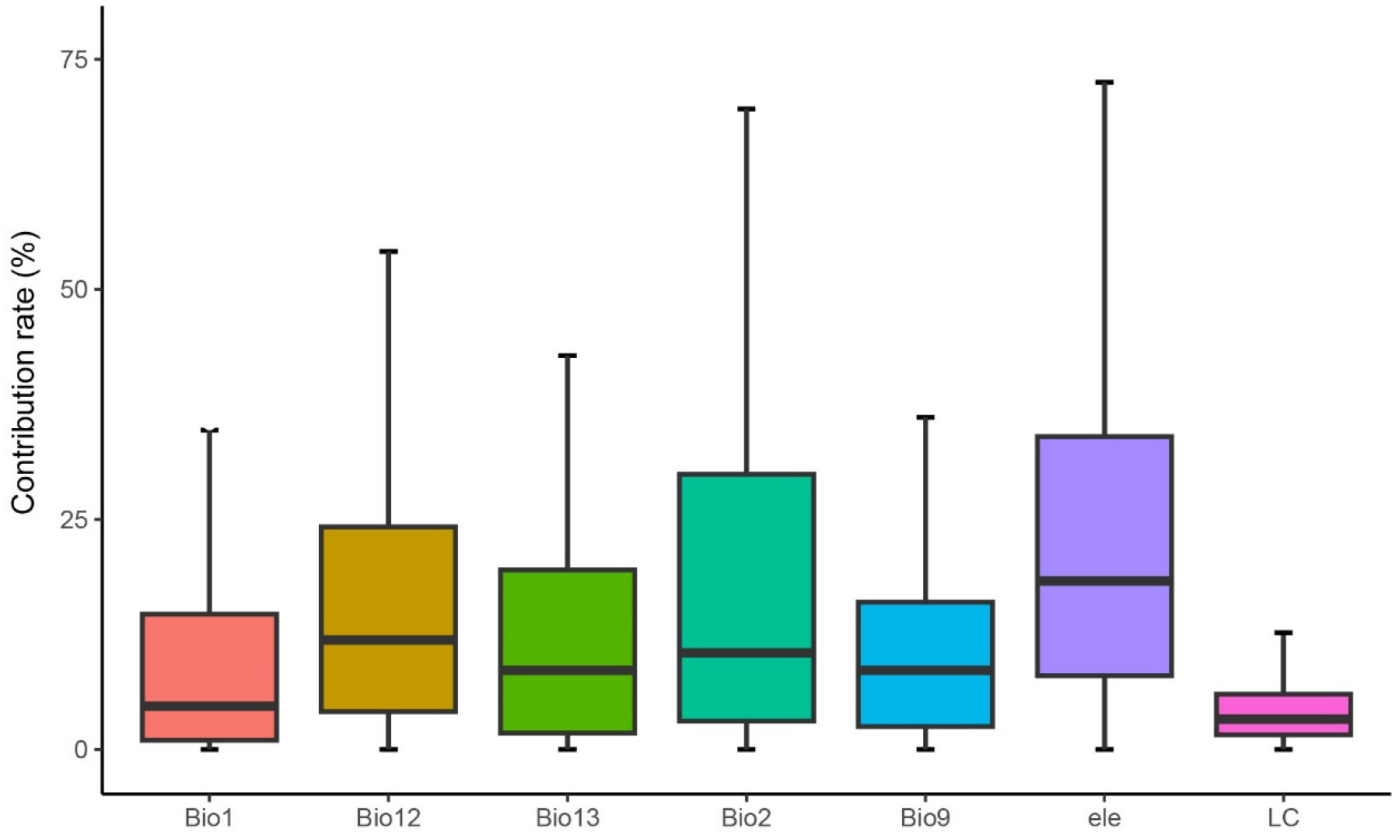
Contribution rate of environmental factors. Bio1, annual mean temperature; Bio2, mean diurnal range/ temperature annual range; Bio9, mean temperature of driest quarter; Bio12, annual precipitation; Bio13, precipitation of wettest month; ele: altitude; LC, land cover.

The average AUC values for the training set and validation set of all species model results are 0.91 ± 0.07 and 0.85 ± 0.09, respectively. The model exhibits good predictive accuracy with strong reliability. The average contribution of each environmental factor is shown in Figure 1, with the higher contributing factors being elevation (24.75%), bio2 (18.08%), and seasonal variation in precipitation (17.78%). This indicates that these factors are important drivers influencing the spatial distribution of bird species in Jiangsu Province.

However, there are significant variations in the contribution of different environmental factors among species. For example, land cover type primarily affects species such as the Crested Serpent Eagle (*Spilornis cheela*), Oriental Honey Buzzard (*Pernis ptilorhynchus*), White‐crowned Forktail (*Enicurus leschenaulti*), and Besra (*Accipiter virgatus*). Elevation mainly influences species like the Himalayan Lark (*Dendronanthus indicus*), Eurasian Hobby (*Falco subbuteo*), and Large Hawk Cuckoo (*Hierococcyx sparverioides*).

### 3.3 Species richness

**FIGURE 2:**
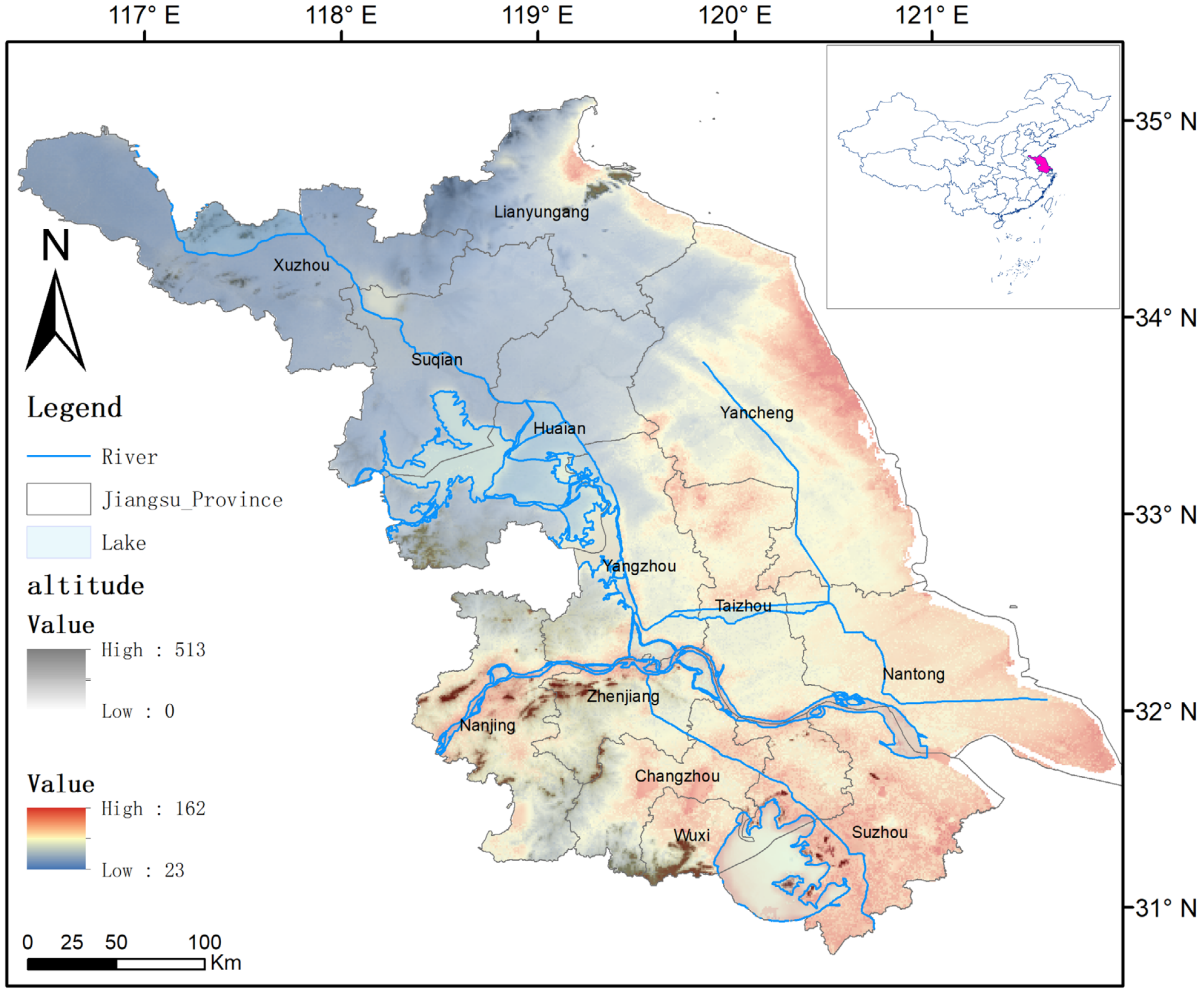
Predicted species richness of birds in Jiangsu Province, China (*n* = 312). The inset shows the black area illustrates the apparent association of richness with the hydrological network and/or elevation.

**FIGURE 3:**
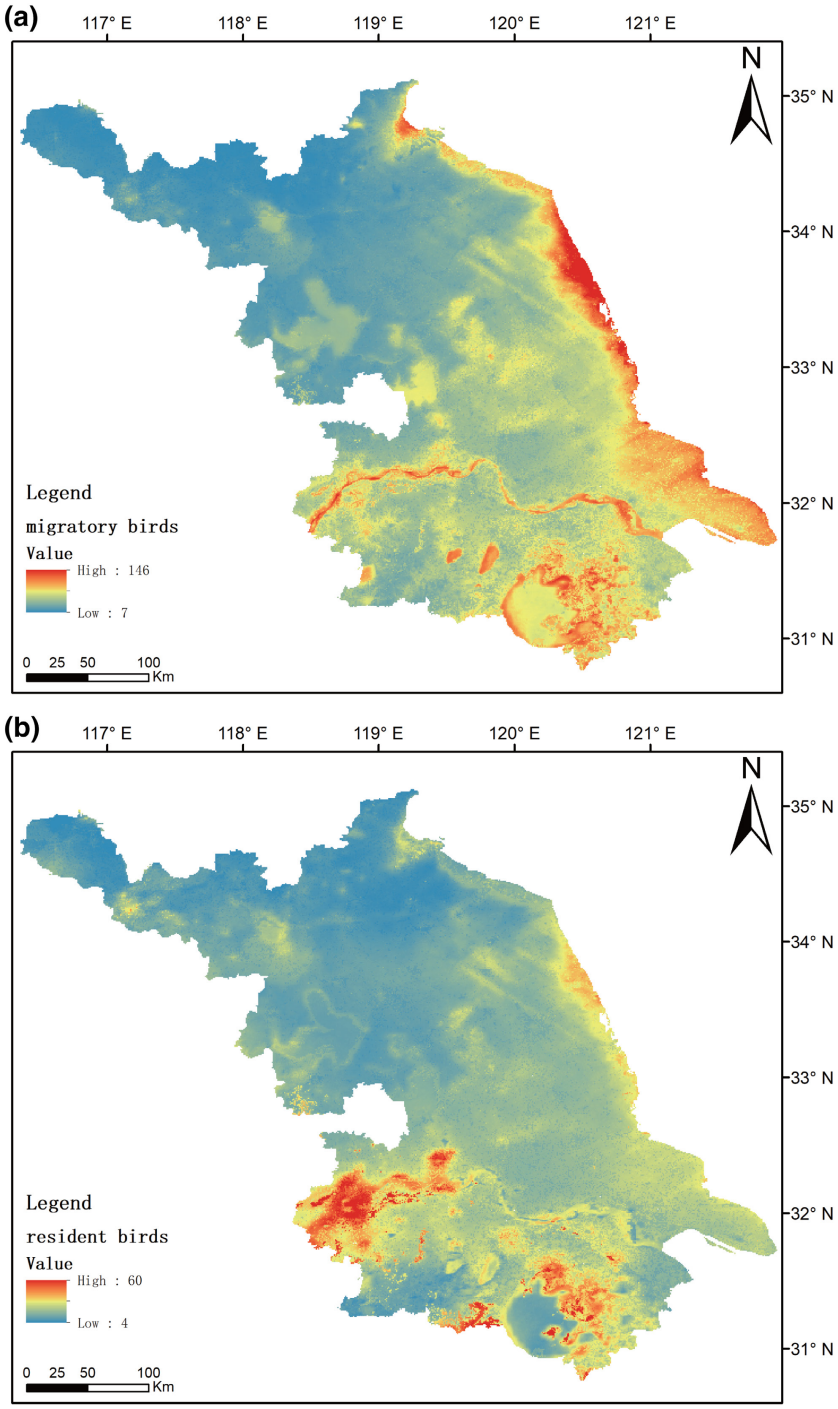
Comparative analysis of the potential spatial distribution differences in species richness between migratory (*n* = 237) and resident (*n* = 75) birds in Jiangsu Province, China. (a) migratory birds, (b) resident birds.

Based on the potential distribution of bird species in Jiangsu Province, this study identified a clear distribution pattern, characterized by a higher species richness in the south, lower in the north, and higher in the east, lower in the west (Figure 2). The regions with relatively concentrated bird distribution are mainly three areas: the eastern coastal region, the surrounding area of the lower reaches of the Yangtze River, and the surrounding area of Taihu Lake. The distribution patterns of migratory birds and resident birds are similar to the overall bird distribution pattern. However, it is worth noting that migratory birds are more concentrated in the coastal areas and the Yangtze River basin, while resident birds are concentrated in the Taihu Lake region and the southwest corner of Jiangsu Province (Figure 3). The diversity of resident birds in the middle and lower reaches of the Yangtze River in Jiangsu is significantly higher than in the eastern coastal areas, while migratory birds exhibit higher diversity in the eastern coastal areas compared to the middle and lower reaches of the Yangtze River.

**FIGURE 4:**
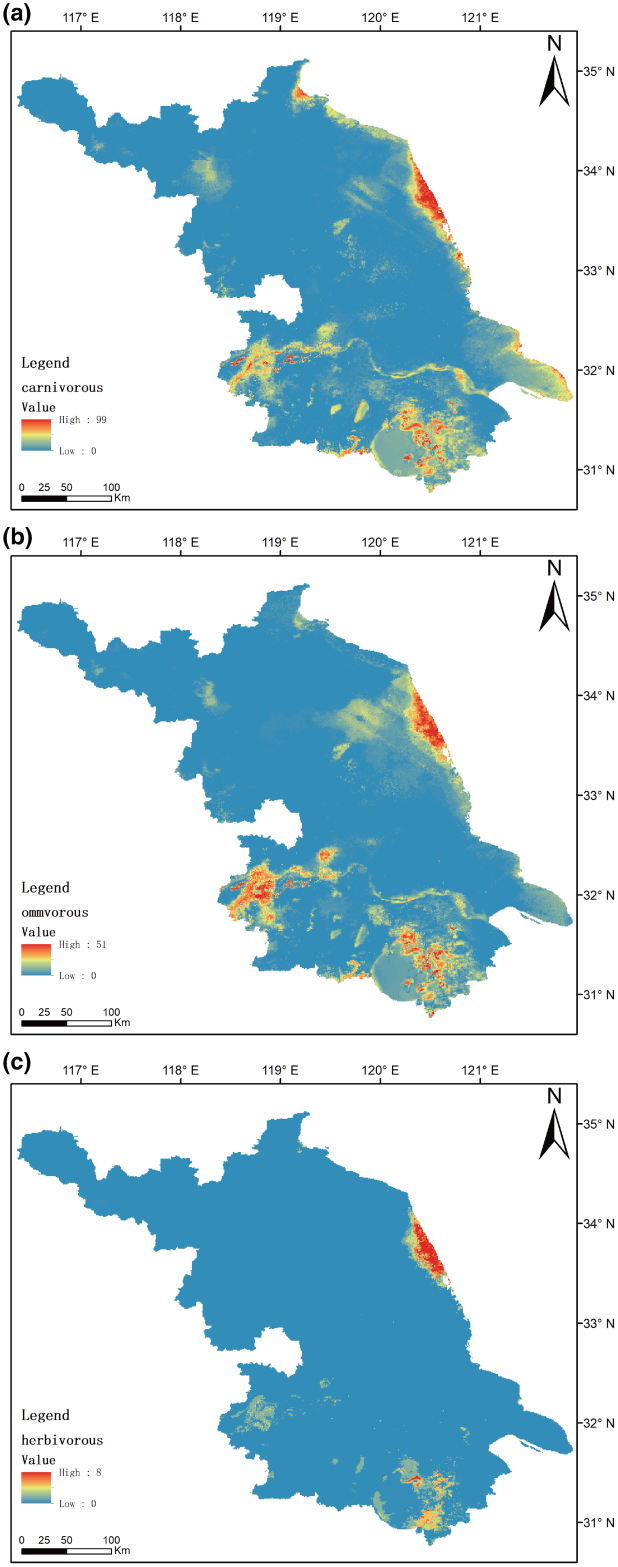
The impact of dietary types on the potential spatial distribution of species richness in Jiangsu Province, China. (a) carnivorous birds (*n* = 196), (b) omnivorous birds (*n* = 105), (c) herbivorous birds (*n* = 11).

From the perspective of feeding habits, the potential distribution areas of carnivorous and omnivorous birds are relatively extensive, while the distribution area of herbivorous birds is smaller than others (Figure 4). The patterns of carnivorous and omnivorous birds are similar to the overall bird distribution pattern, with the main difference being the smaller areas of moderate richness. Regions with a high richness of herbivorous bird species are primarily concentrated in the eastern coastal areas, with only a small portion in the Taihu region and common river basins, primarily in areas of moderate richness.

**FIGURE 5:**
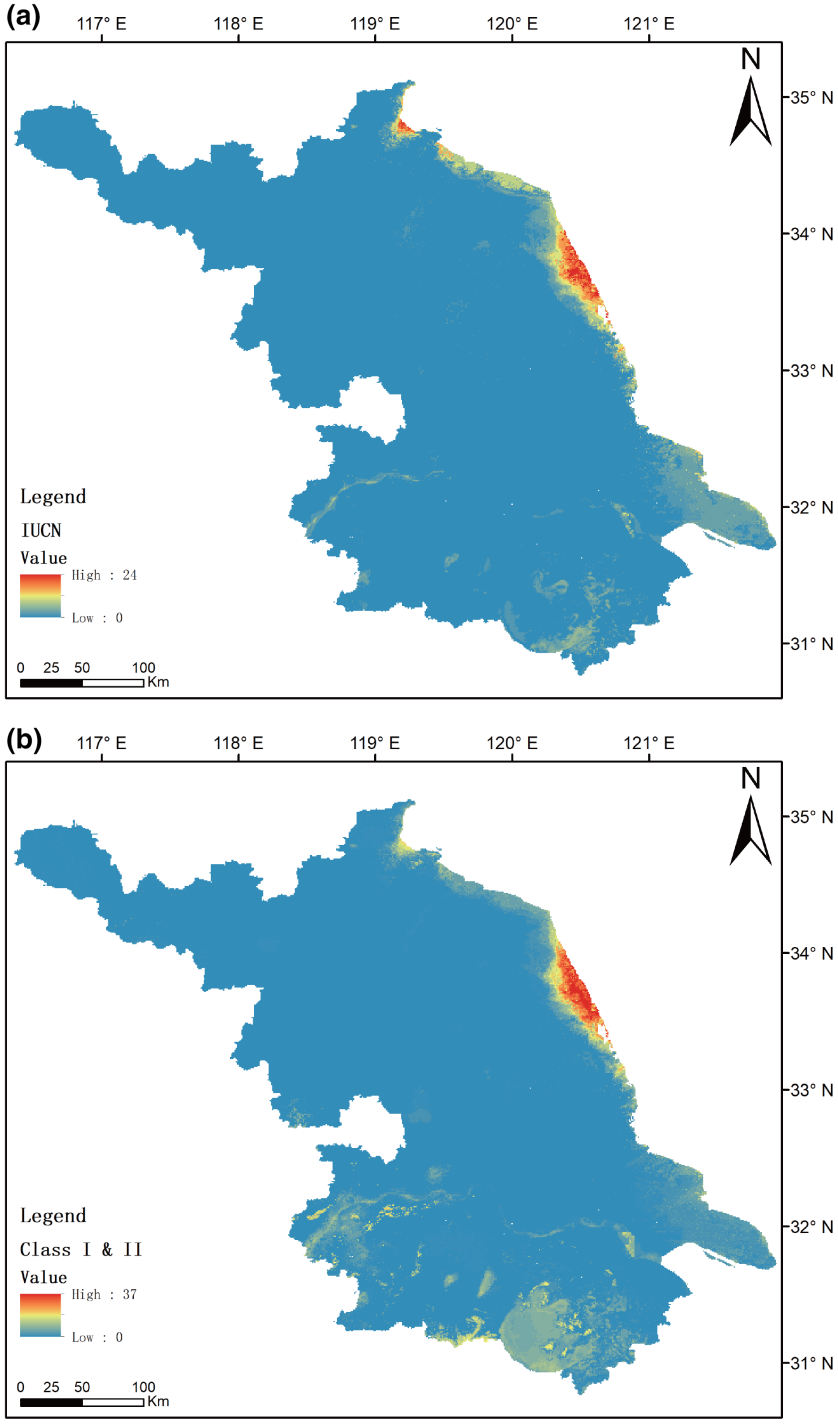
The potential spatial distribution of species richness for bird species listed on the IUCN Red List (*n* = 33) and those specifically protected in China (*n* = 63).

The potential distribution areas of nationally protected bird species and threatened bird species in China show the highest richness in the eastern coastal areas, with moderate richness in the middle and lower reaches of the Yangtze River and the Taihu Lake region (Figure 5, Appendix Figure S2).

### 3.4 Hotspot distribution and GAP analysis

**FIGURE 6:**
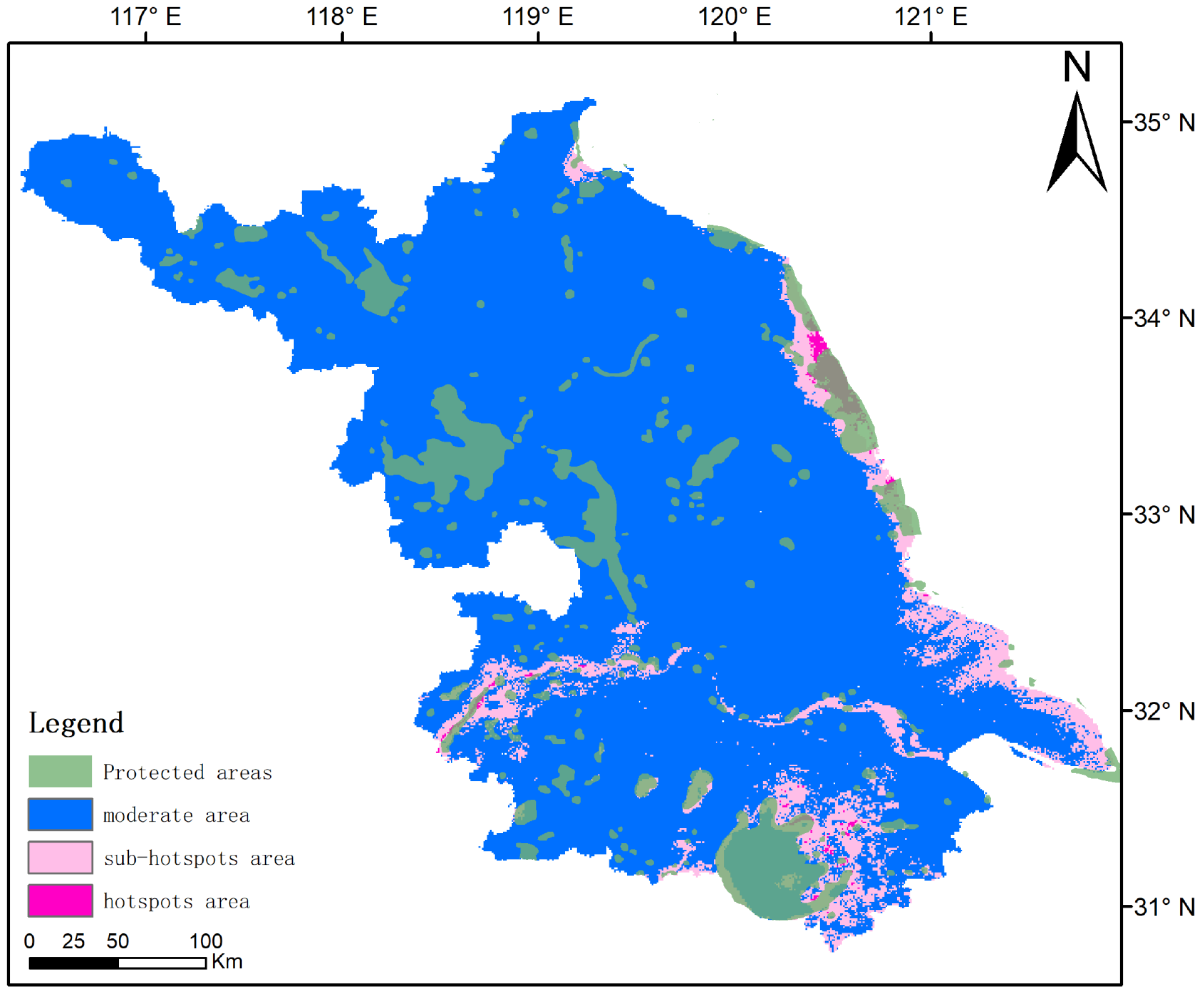
Maps showing the current protected areas of Jiangsu Province, China, the potential avifauna hotspots areas identified in our spatial analysis.

Using the MaxEnt model to analyze the bird diversity hotspots in Jiangsu Province (Figure 6), the results indicate that the bird hotspot areas cover an area of 1420.28 km^2^, while sub‐hotspot areas cover 3367.11 km^2^. The distribution of nature protection zones in Jiangsu covers 51.55% of the hotspot areas (732.09 km^2^) and 25.12% of the sub‐hotspot areas (845.69 km^2^). However, there are still 688.19 km^2^ of hotspot areas and 2521.42 km^2^ of sub‐hotspot areas outside the nature protection zones (Appendix Figure S3).

## 4 DISCUSSION

In this study, we create models showing the distribution of species richness and rarity using SDM for almost all known bird species in the rapidly urbanizing province of Jiangsu, China. We used the MaxEnt model to identify suitable habitat distribution areas for avian species, explore the relationship between environmental variables, and evaluate the degree of influence of each environmental parameter on the bird distribution. Birds play crucial roles in ecosystem functioning, such as dispersion, pollination, and plant reproduction, and serve as vital indicators of landscape conditions (Fraixedas et al., 2020), so they are frequently utilized by scientists, decision makers, and non‐governmental organizations to highlight and promote conservation policies and needs (Chan et al., 2019). To ensure efficient bird resource protection, we simulate priority habitats and conducted a comprehensive analysis to select the priority conservation areas. The present study provides a conservation modeling framework that can be used by policymakers to assess the efficiency of natural PAs and optimize their existing ones.

In this study, we identified three hotspots in Jiangsu Province, such as the eastern coastal region, the surrounding area of the lower reaches of the Yangtze River, and the surrounding area of Taihu Lake. Among the bird species involved in this modeling, the Passeriformes order has the highest number of species in the avian community, with 133 species in 31 families, while the Charadriiformes order dominates among waterbirds, comprising 62 species in 7 families. Jiangsu Province is located along the East Asian–Australasian Flyway (EAAF), which is one of the world's most crucial flyways for waterbirds, providing millions of waterbirds with locations for replenishment, breeding, and overwintering (Duan et al., 2020). Jiangsu Province supports a diverse avian population of 468 species, accounting for about 29.62% of the China's (1580 species) bird species (Zheng, 2017). In recent decades, Jiangsu Province has experienced rapid economic development. With the increase in urban population, rapid and sustained urbanization may lead to significant changes in the ecosystem functions of cities (Plummer et al., 2020; Yang et al., 2020), inevitably affecting the survival, distribution, and growth of birds. Enhancing our understanding of bird distribution in Jiangsu Province is crucial for developing and implementing timely conservation strategies.

Moreover, the coast of Jiangsu Province serves as a crucial habitat for molting and overwintering waterbirds (Sun et al., 2021). For example, 21 species that meet the Ramsar listing criterion of 1% population level were observed at Tiaozini, located on the southern Jiangsu coast (Bai et al., 2015); furthermore, the northern Jiangsu coast also hosts 26 species on its Lianyungang coast that meet the criterion (Bai et al., 2015; Chan et al., 2019). Detailed data on species diversity and the rarity of range sizes within this extensive taxonomic group also offer exciting opportunities for macroecological research across various spatial scales. Enhanced understanding of avifauna regional geographic distribution is essential for developing timely conservation strategies.

However, there is relatively limited research on avifauna in the Nanjing–Zhenjiang riverside region and inland lake areas. Nanjing–Zhenjiang hotspot encompasses various wetland and urban parks within the Yangtze River basin. Protection gaps in Nanjing are mainly found south of the Yangtze River, from Niushoushan to the west of Qixia Mountain, and on the north side, from Nanjing Pearl Spring Scenic Area to Donglingyan Mountain Scenic Area. In Zhenjiang, protection gap areas are concentrated in Runzhou District, while in Yangzhou, they are focused in Kanjiang District and Guangling District. In Jiangsu Province, there are two major freshwater lakes, Hongze Lake and Taihu Lake, which form crucial wetland ecosystems essential for biodiversity. These lakes serve as important wintering grounds for waterfowl species during the winter, and they also function as crucial habitats for endangered species such as cranes, raptors, and other birds. However, there is a need for more comprehensive research on inland lake ecosystems.

The detailed data on species diversity within this taxonomic group, which offers promising opportunities for macroecological research across various spatial scales, involve a challenging process of collecting the necessary detailed and high‐quality occurrence data (Herkt et al., 2016). However, a few studies have utilized SDM to encompass broad areas with high spatial detail and include a diverse set of species (Hu et al., 2017). The lack of this knowledge often hinders the effective planning and execution of conservation efforts at the appropriate spatial levels (Stevens & Conway, 2020b). Furthermore, it constrains advancements in macroecology, as the spatial structure of range size and species diversity patterns may vary at more detailed scales (Carroll et al., 2023; Stevens & Conway, 2020a).

## 5 CONCLUSIONS

Our study, focusing on birds as a representative taxonomic group, employs the MaxEnt model and extensive occurrence data to create species distribution models (SDMs) for 312 bird species at a fine resolution of 1 km^2^ in Jiangsu Province. The contribution analysis indicated the significant role of altitude bio2 and bi12 in shaping bird species distribution and diversity. Our findings identified three primary areas with high potential bird species richness: the eastern coastal region, the surrounding area of the lower reaches of the Yangtze River, and the surrounding area of Taihu Lake. While most hotspots align with existing protected areas, some regions, such as coastal areas of Sheyang County in Yancheng City, as well as portions along the Yangtze River in Nanjing and Zhenjiang, lack sufficient protection. The study highlights the significance of future conservation efforts in mitigating biodiversity loss, especially the risks to endangered species.

## AUTHOR CONTRIBUTIONS

**Wan Chen:** Conceptualization (equal); data curation (equal); formal analysis (equal); software (equal); writing – original draft (equal). **Xuan Wang:** Data curation (equal); formal analysis (equal). **Yuanyuan Cai:** Formal analysis (equal); software (equal). **Xinglong Huang:** Data curation (equal); investigation (equal). **Peng Li:** Software (equal); writing – original draft (equal). **Wei Liu:** Resources (equal); validation (equal). **Qing Chang:** Funding acquisition (equal); writing – original draft (equal). **Chaochao Hu:** Conceptualization (equal); data curation (equal); methodology (equal); software (equal); writing – original draft (equal).

## FUNDING INFORMATION

This research was funded by Wildlife Resources Monitoring Project of Important Wetland in Jiangsu Province, National Forestry Reform and Development Fund of China, Natural Science Research of Jiangsu Higher Education Institutions of China (grant number 20KJD180004); Jiangsu Agricultural Biodiversity Cultivation and Utilization Research Center, grant number 100605‐2023‐KY‐00335; and the Jiangsu Academy of Forestry Youth Foundation [JAF‐2022‐01]. The funders had no role in study design, data collection and analysis, decision to publish, or preparation of the manuscript.

## CONFLICT OF INTEREST STATEMENT

The authors declare no conflict of interest.

## Supporting information


Appendix S1.

## Data Availability

The authors confirm that the data supporting the findings of this study are available within the article and/or its supplementary materials. Interested readers to other material could to request them from the corresponding author.
