# Supplementary material for: Potential distribution patterns and species richness of avifauna in rapidly urbanizing East China
Source: Ecol Evol. 2024 Jun 18;14(6):e11515. doi: 10.1002/ece3.11515 (PMC11183928; doi:10.1002/ece3.11515)
Supplement: Supplementary file 1 — Appendix S1. [file ECE3-14-e11515-s001.docx]

## Appendix:

**Figure S1.** Pearson correlations of 19 bioclimatic variables. The variables with correlation coefficients of more than 0.7 were excluded.

**Figure S2.** Potential spatial distribution maps of bird species based on threatened bird in Jiangsu Province, China. (a) CR (Critically Endangered) and EN (Endangered) birds, (b) NT (Near Threatened), (c) VU (Vulnerable).

**Figure S3.** Potential distribution hotspots of bird species and their relationship with rivers and altitude map in Jiangsu Province, China.

**Table S1:** Environmental factors used in species distribution models.

**Table S2:** Data used in this study.

## Figure S1


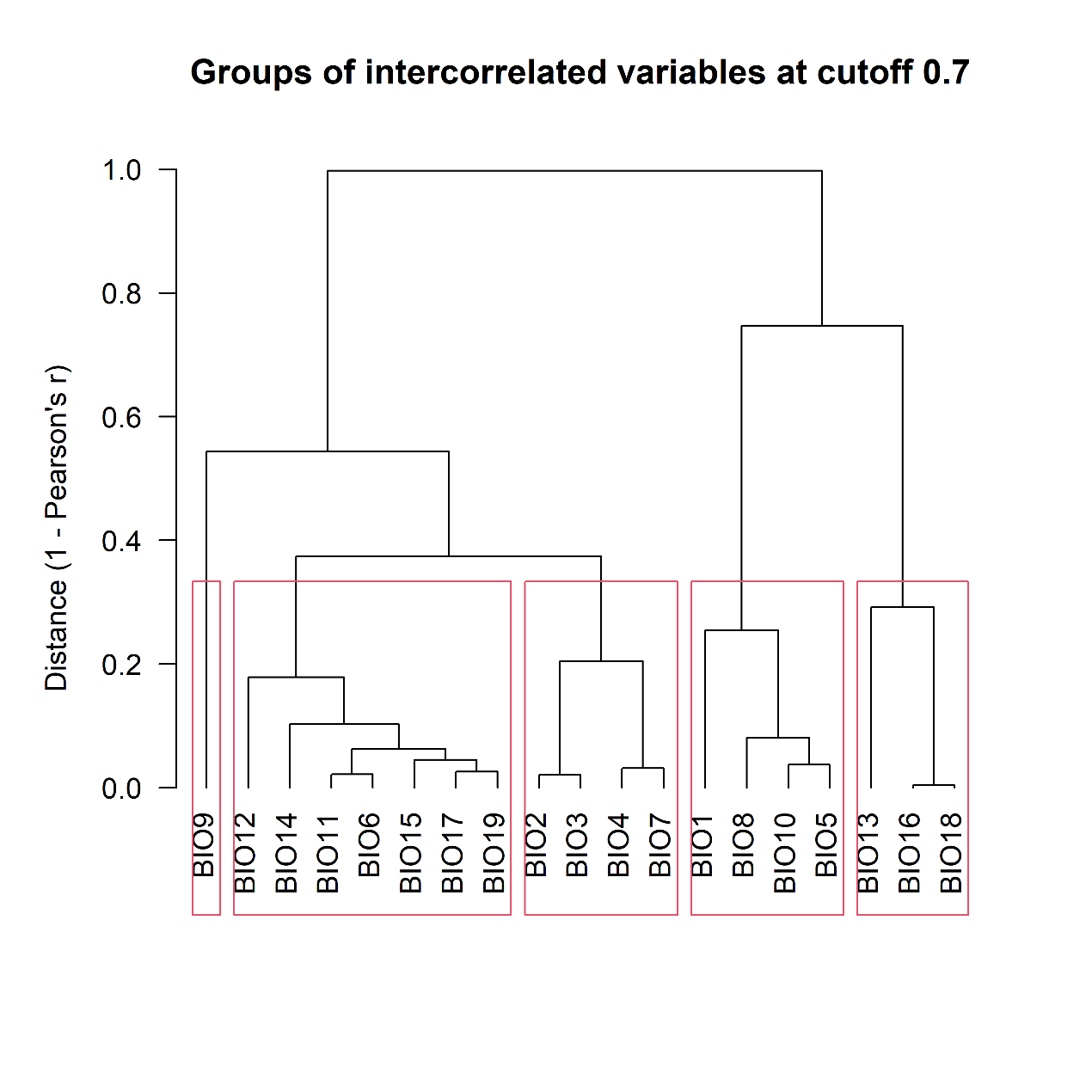


## Figure S2


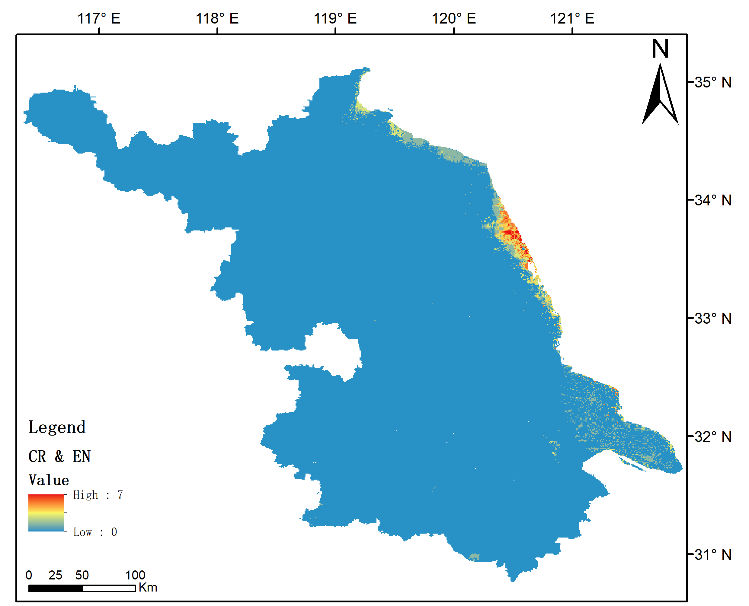


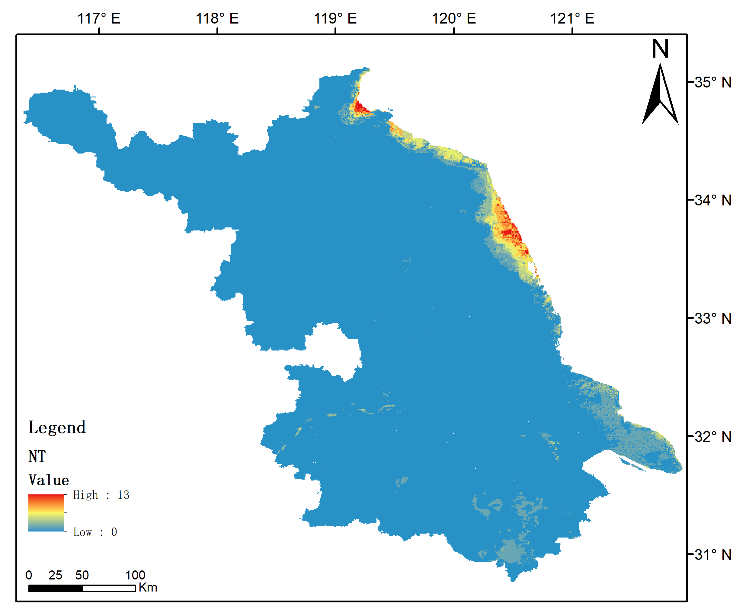


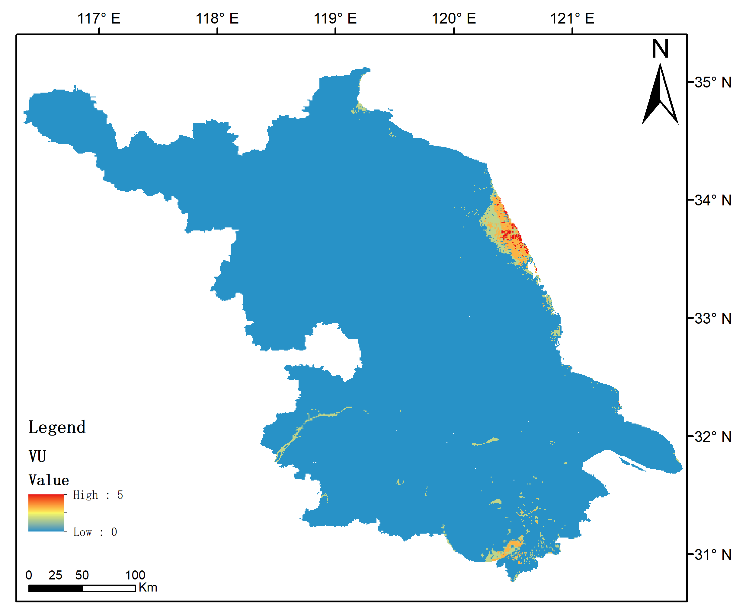


## Figure S3


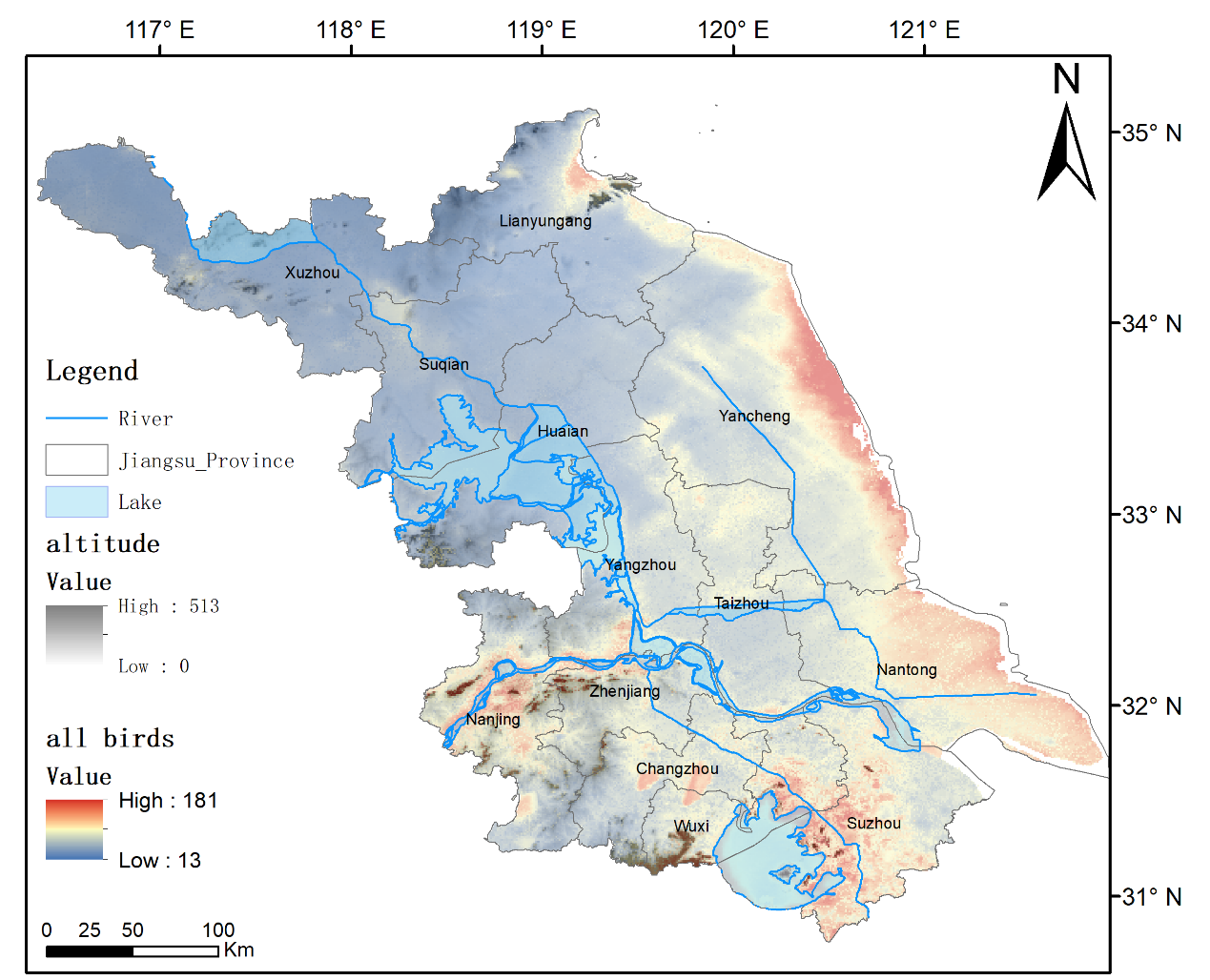


## Table S1 Environmental factors used in species distribution models

| Environmental variable | Description | Unit |
| --- | --- | --- |
| Bio1 | Annual Mean Temperature | ℃ |
| Bio2 | Mean Diurnal Range | ℃ |
| Bio3 | Isothermality | ℃ |
| Bio4 | Temperature Seasonality | ℃ |
| Bio5 | Max Temperature of Warmest Month | ℃ |
| Bio6 | Min Temperature of Coldest Month | ℃ |
| Bio7 | Temperature Annual Range | ℃ |
| Bio8 | Mean Temperature of Wettest Quarter | ℃ |
| Bio9 | Mean Temperature of Driest Quarter | ℃ |
| Bio10 | Mean Temperature of Warmest Quarter | ℃ |
| Bio11 | Mean Temperature of Coldest Quarter | ℃ |
| Bio12 | Annual Precipitation | mm |
| Bio13 | Precipitation of Wettest Month | mm |
| Bio14 | Precipitation of Driest Month | mm |
| Bio15 | Precipitation Seasonality | mm |
| Bio16 | Precipitation of Wettest Quarter | mm |
| Bio17 | Precipitation of Driest Quarter | mm |
| Bio18 | Precipitation of Warmest Quarter | mm |
| Bio19 | Precipitation of Coldest Quarter | mm |
| Elev1 | Altitude | m |
| LC | Land cover | — |

## Table S2: Data used in this study.

| English name | Scientific name | Order | Family | Migration | IUCN | Trophic.Level | Protection level |
| --- | --- | --- | --- | --- | --- | --- | --- |
| Japanese Quail | *Coturnix japonica* | Galliformes | Phasianidae | W | NT | Omnivore |  |
| Chinese Bamboo Partridge | *Bambusicola thoracicus* | Galliformes | Phasianidae | R | LC | Omnivore |  |
| Common Pheasant | *Phasianus colchicus* | Galliformes | Phasianidae | R | LC | Omnivore |  |
| Swan Goose | *Anser cygnoid* | Anseriformes | Anatidae | W | VU | Herbivore | II |
| Bean Goose | *Anser fabalis* | Anseriformes | Anatidae | W | LC | Herbivore |  |
| Tundra Bean Goose | *Anser serrirostris* | Anseriformes | Anatidae | W | NR | Herbivore |  |
| Graylag Goose | *Anser anser* | Anseriformes | Anatidae | W | LC | Omnivore |  |
| Greater White-fronted Goose | *Anser albifrons* | Anseriformes | Anatidae | W | LC | Herbivore | II |
| Mute Swan | *Cygnus olor* | Anseriformes | Anatidae | W | LC | Omnivore | II |
| Tundra Swan | *Cygnus columbianus* | Anseriformes | Anatidae | W | LC | Herbivore | II |
| Common Shelduck | *Tadorna tadorna* | Anseriformes | Anatidae | W | LC | Omnivore |  |
| Ruddy Shelduck | *Tadorna ferruginea* | Anseriformes | Anatidae | W | LC | Omnivore |  |
| Mandarin Duck | *Aix galericulata* | Anseriformes | Anatidae | W | LC | Omnivore | II |
| Gadwall | *Mareca strepera* | Anseriformes | Anatidae | W | LC | Herbivore |  |
| Falcated Duck | *Mareca falcata* | Anseriformes | Anatidae | W | NT | Herbivore |  |
| Eurasian Wigeon | *Mareca penelope* | Anseriformes | Anatidae | W | LC | Herbivore |  |
| Mallard | *Anas platyrhynchos* | Anseriformes | Anatidae | W | LC | Omnivore |  |
| Eastern Spot-billed Duck | *Anas zonorhyncha* | Anseriformes | Anatidae | W | LC | Omnivore |  |
| Northern Pintail | *Anas acuta* | Anseriformes | Anatidae | W | LC | Omnivore |  |
| Green-winged Teal | *Anas crecca* | Anseriformes | Anatidae | W | LC | Omnivore |  |
| Northern Shoveler | *Spatula clypeata* | Anseriformes | Anatidae | W | LC | Omnivore |  |
| Garganey | *Spatula querquedula* | Anseriformes | Anatidae | P | LC | Omnivore |  |
| Baikal Teal | *Sibirionetta formosa* | Anseriformes | Anatidae | W | LC | Omnivore | II |
| Common Pochard | *Aythya ferina* | Anseriformes | Anatidae | W | VU | Omnivore |  |
| Baer's Pochard | *Aythya baeri* | Anseriformes | Anatidae | W | CR | Omnivore | I |
| Ferruginous Duck | *Aythya nyroca* | Anseriformes | Anatidae | W | NT | Omnivore |  |
| Tufted Duck | *Aythya fuligula* | Anseriformes | Anatidae | W | LC | Carnivore |  |
| Greater Scaup | *Aythya marila* | Anseriformes | Anatidae | W | LC | Omnivore |  |
| Smew | *Mergellus albellus* | Anseriformes | Anatidae | W | LC | Carnivore | II |
| Common Merganser | *Mergus merganser* | Anseriformes | Anatidae | W | LC | Carnivore |  |
| Red-breasted Merganser | *Mergus serrator* | Anseriformes | Anatidae | W | LC | Carnivore |  |
| Little Grebe | *Tachybaptus ruficollis* | Podicipediformes | Podicipedidae | R | LC | Carnivore |  |
| Great Crested Grebe | *Podiceps cristatus* | Podicipediformes | Podicipedidae | W | LC | Carnivore |  |
| Black-necked Grebe | *Podiceps nigricollis* | Podicipediformes | Podicipedidae | W | LC | Carnivore | II |
| Rock Pigeon | *Columba livia* | Columbiformes | Columbidae | R | LC | Herbivore |  |
| Oriental Turtle Dove | *Streptopelia orientalis* | Columbiformes | Columbidae | R | LC | Omnivore |  |
| Red Turtle Dove | *Streptopelia tranquebarica* | Columbiformes | Columbidae | R | LC | Omnivore |  |
| Spotted Dove | *Streptopelia chinensis* | Columbiformes | Columbidae | R | LC | Omnivore |  |
| Grey Nightjar | *Caprimulgus indicus* | Caprimulgiformes | Caprimulgidae | S | LC | Carnivore |  |
| Himalayan Swiftlet | *Aerodramus brevirostris* | Caprimulgiformes | Apodidae | S | LC | Carnivore |  |
| White-throated Needletail | *Hirundapus caudacutus* | Caprimulgiformes | Apodidae | P | LC | Carnivore |  |
| Fork-tailed Swift | *Apus pacificus* | Caprimulgiformes | Apodidae | S | LC | Carnivore |  |
| Lesser Coucal | *Centropus bengalensis* | Cuculiformes | Cuculidae | R | LC | Carnivore | II |
| Chestnut-winged Cuckoo | *Clamator coromandus* | Cuculiformes | Cuculidae | S | LC | Carnivore |  |
| Common Koel | *Eudynamys scolopaceus* | Cuculiformes | Cuculidae | S | LC | Omnivore |  |
| Large Hawk Cuckoo | *Hierococcyx sparverioides* | Cuculiformes | Cuculidae | S | LC | Carnivore |  |
| Lesser Cuckoo | *Cuculus poliocephalus* | Cuculiformes | Cuculidae | S | LC | Carnivore |  |
| Indian Cuckoo | *Cuculus micropterus* | Cuculiformes | Cuculidae | S | LC | Carnivore |  |
| Common Cuckoo | *Cuculus canorus* | Cuculiformes | Cuculidae | S | LC | Carnivore |  |
| Brown-cheeked Rail | *Rallus indicus* | Gruiformes | Rallidae | W | LC | Omnivore |  |
| Brown Crake | *Zapornia akool* | Gruiformes | Rallidae | R | LC | Omnivore |  |
| White-breasted Waterhen | *Amaurornis phoenicurus* | Gruiformes | Rallidae | S | LC | Omnivore |  |
| Common Moorhen | *Gallinula chloropus* | Gruiformes | Rallidae | S | LC | Omnivore |  |
| Common Coot | *Fulica atra* | Gruiformes | Rallidae | W | LC | Omnivore |  |
| Sandhill Crane | *Grus canadensis* | Gruiformes | Gruidae |  | LC | Omnivore | II |
| Red-crowned Crane | *Grus japonensis* | Gruiformes | Gruidae | W | EN | Omnivore | I |
| Common Crane | *Grus grus* | Gruiformes | Gruidae | W | LC | Omnivore | II |
| Hooded Crane | *Grus monacha* | Gruiformes | Gruidae | W | VU | Omnivore | I |
| Eurasian Oystercatcher | *Haematopus ostralegus* | Charadriiformes | Haematopodidae | W | NT | Carnivore |  |
| Black-winged Stilt | *Himantopus himantopus* | Charadriiformes | Recurvirostridae | P | LC | Carnivore |  |
| Pied Avocet | *Recurvirostra avosetta* | Charadriiformes | Recurvirostridae | W | LC | Carnivore |  |
| Northern Lapwing | *Vanellus vanellus* | Charadriiformes | Charadriidae | W | NT | Omnivore |  |
| Grey-headed Lapwing | *Vanellus cinereus* | Charadriiformes | Charadriidae | S | LC | Omnivore |  |
| Pacific Golden Plover | *Pluvialis fulva* | Charadriiformes | Charadriidae | P | LC | Carnivore |  |
| Grey Plover | *Pluvialis squatarola* | Charadriiformes | Charadriidae | P | LC | Carnivore |  |
| Long-billed Plover | *Charadrius placidus* | Charadriiformes | Charadriidae | P | LC | Omnivore |  |
| Little Ringed Plover | *Charadrius dubius* | Charadriiformes | Charadriidae | S | LC | Carnivore |  |
| Kentish Plover | *Charadrius alexandrinus* | Charadriiformes | Charadriidae | R | LC | Carnivore |  |
| Lesser Sand Plover | *Charadrius mongolus* | Charadriiformes | Charadriidae | P | LC | Carnivore |  |
| Greater Sand Plover | *Charadrius leschenaultii* | Charadriiformes | Charadriidae | P | LC | Carnivore |  |
| Oriental Plover | *Charadrius veredus* | Charadriiformes | Charadriidae | P | LC | Carnivore |  |
| Pheasant-tailed Jacana | *Hydrophasianus chirurgus* | Charadriiformes | Jacanidae | S | LC | Omnivore | II |
| Eurasian Woodcock | *Scolopax rusticola* | Charadriiformes | Scolopacidae | W | LC | Omnivore |  |
| Pintail Snipe | *Gallinago stenura* | Charadriiformes | Scolopacidae | P | LC | Carnivore |  |
| Common Snipe | *Gallinago gallinago* | Charadriiformes | Scolopacidae | W | LC | Carnivore |  |
| Asian Dowitcher | *Limnodromus semipalmatus* | Charadriiformes | Scolopacidae | P | NT | Carnivore | II |
| Black-tailed Godwit | *Limosa limosa* | Charadriiformes | Scolopacidae | P | NT | Carnivore |  |
| Bar-tailed Godwit | *Limosa lapponica* | Charadriiformes | Scolopacidae | P | NT | Carnivore |  |
| Little Curlew | *Numenius minutus* | Charadriiformes | Scolopacidae | P | LC | Omnivore | II |
| Whimbrel | *Numenius phaeopus* | Charadriiformes | Scolopacidae | P | LC | Carnivore |  |
| Eurasian Curlew | *Numenius arquata* | Charadriiformes | Scolopacidae | P | NT | Carnivore | II |
| Eastern Curlew | *Numenius madagascariensis* | Charadriiformes | Scolopacidae | P | EN | Carnivore | II |
| Spotted Redshank | *Tringa erythropus* | Charadriiformes | Scolopacidae | P | LC | Carnivore |  |
| Common Redshank | *Tringa totanus* | Charadriiformes | Scolopacidae | W | LC | Carnivore |  |
| Marsh Sandpiper | *Tringa stagnatilis* | Charadriiformes | Scolopacidae | P | LC | Carnivore |  |
| Common Greenshank | *Tringa nebularia* | Charadriiformes | Scolopacidae | W | LC | Carnivore |  |
| Nordmann's Greenshank | *Tringa guttifer* | Charadriiformes | Scolopacidae | P | EN | Carnivore | I |
| Green Sandpiper | *Tringa ochropus* | Charadriiformes | Scolopacidae | W | LC | Carnivore |  |
| Wood Sandpiper | *Tringa glareola* | Charadriiformes | Scolopacidae | P | LC | Carnivore |  |
| Grey-tailed Tattler | *Tringa brevipes* | Charadriiformes | Scolopacidae | P | NT | Carnivore |  |
| Terek Sandpiper | *Xenus cinereus* | Charadriiformes | Scolopacidae | P | LC | Carnivore |  |
| Common Sandpiper | *Actitis hypoleucos* | Charadriiformes | Scolopacidae | W | LC | Carnivore |  |
| Ruddy Turnstone | *Arenaria interpres* | Charadriiformes | Scolopacidae | P | LC | Omnivore | II |
| Great Knot | *Calidris tenuirostris* | Charadriiformes | Scolopacidae | P | EN | Carnivore | II |
| Red Knot | *Calidris canutus* | Charadriiformes | Scolopacidae | P | NT | Omnivore |  |
| Sanderling | *Calidris alba* | Charadriiformes | Scolopacidae | P | LC | Omnivore |  |
| Red-necked Stint | *Calidris ruficollis* | Charadriiformes | Scolopacidae | P | NT | Carnivore |  |
| Spoon-billed Sandpiper | *Calidris pygmeus* | Charadriiformes | Scolopacidae | P | CR | Carnivore | I |
| Temminck's Stint | *Calidris temminckii* | Charadriiformes | Scolopacidae | P | LC | Carnivore |  |
| Long-toed Stint | *Calidris subminuta* | Charadriiformes | Scolopacidae | P | LC | Carnivore |  |
| Sharp-tailed Sandpiper | *Calidris acuminata* | Charadriiformes | Scolopacidae | P | LC | Carnivore |  |
| Broad-billed Sandpiper | *Calidris falcinellus* | Charadriiformes | Scolopacidae | P | LC | Carnivore | II |
| Ruff | *Calidris pugnax* | Charadriiformes | Scolopacidae | P | LC | Carnivore |  |
| Curlew Sandpiper | *Calidris ferruginea* | Charadriiformes | Scolopacidae | P | NT | Carnivore |  |
| Dunlin | *Calidris alpina* | Charadriiformes | Scolopacidae | W | LC | Carnivore |  |
| Red-necked Phalarope | *Phalaropus lobatus* | Charadriiformes | Scolopacidae | P | LC | Carnivore |  |
| Oriental Pratincole | *Glareola maldivarum* | Charadriiformes | Glareolidae | S | LC | Carnivore |  |
| Black-headed Gull | *Chroicocephalus ridibundus* | Charadriiformes | Laridae | W | LC | Carnivore |  |
| Saunders's Gull | *Saundersilarus saundersi* | Charadriiformes | Laridae | P | VU | Carnivore | I |
| Relict Gull | *Ichthyaetus relictus* | Charadriiformes | Laridae | P | VU | Carnivore | I |
| Black-tailed Gull | *Larus crassirostris* | Charadriiformes | Laridae | P | LC | Carnivore |  |
| Mew Gull | *Larus canus* | Charadriiformes | Laridae | W | LC | Carnivore |  |
| Lesser Black-backed Gull | *Larus fuscus* | Charadriiformes | Laridae | W | LC | Carnivore |  |
| Siberian Gull | *Larus smithsonianus* | Charadriiformes | Laridae | W | LC | Carnivore |  |
| Gull-billed Tern | *Gelochelidon nilotica* | Charadriiformes | Laridae | P | LC | Carnivore |  |
| Caspian Tern | *Hydroprogne caspia* | Charadriiformes | Laridae | S | LC | Carnivore |  |
| Little Tern | *Sternula albifrons* | Charadriiformes | Laridae | S | LC | Carnivore |  |
| Common Tern | *Sterna hirundo* | Charadriiformes | Laridae | P | LC | Carnivore |  |
| Whiskered Tern | *Chlidonias hybrida* | Charadriiformes | Laridae | S | LC | Carnivore |  |
| White-winged Tern | *Chlidonias leucopterus* | Charadriiformes | Laridae | S | LC | Carnivore |  |
| Oriental Stork | *Ciconia boyciana* | Ciconiiformes | Ciconiidae | W | EN | Carnivore | I |
| Great Cormorant | *Phalacrocorax carbo* | Sulidae | Phalacrocoracidae | W | LC | Carnivore |  |
| Eurasian Spoonbill | *Platalea leucorodia* | Pelecaniformes | Threskiorothidae | W | LC | Carnivore | II |
| Black-faced Spoonbill | *Platalea minor* | Pelecaniformes | Threskiorothidae | P | EN | Carnivore | I |
| Eurasian Bittern | *Botaurus stellaris* | Pelecaniformes | Ardeidae | W | LC | Carnivore |  |
| Yellow Bittern | *Ixobrychus sinensis* | Pelecaniformes | Ardeidae | S | LC | Carnivore |  |
| Cinnamon Bittern | *Ixobrychus cinnamomeus* | Pelecaniformes | Ardeidae | S | LC | Carnivore |  |
| Black Bittern | *Ixobrychus flavicollis* | Pelecaniformes | Ardeidae | S | LC | Carnivore |  |
| Black-crowned Night Heron | *Nycticorax nycticorax* | Pelecaniformes | Ardeidae | S | LC | Carnivore |  |
| Striated Heron | *Butorides striata* | Pelecaniformes | Ardeidae | S | LC | Carnivore |  |
| Chinese Pond Heron | *Ardeola bacchus* | Pelecaniformes | Ardeidae | R | LC | Carnivore |  |
| Cattle Egret | *Bubulcus ibis* | Pelecaniformes | Ardeidae | R | NR | Carnivore |  |
| Grey Heron | *Ardea cinerea* | Pelecaniformes | Ardeidae | R | LC | Carnivore |  |
| Purple Heron | *Ardea purpurea* | Pelecaniformes | Ardeidae | S | LC | Carnivore |  |
| Great Egret | *Ardea alba* | Pelecaniformes | Ardeidae | S | LC | Carnivore |  |
| Intermediate Egret | *Ardea intermedia* | Pelecaniformes | Ardeidae | S | LC | Carnivore |  |
| Little Egret | *Egretta garzetta* | Pelecaniformes | Ardeidae | S | LC | Carnivore |  |
| Chinese Egret | *Egretta eulophotes* | Pelecaniformes | Ardeidae | S | VU | Carnivore | I |
| Osprey | *Pandion haliaetus* | Accipitriformes | Pandionidae | P | LC | Carnivore | II |
| Black-winged Kite | *Elanus caeruleus* | Accipitriformes | Accipitridae | R | LC | Carnivore | II |
| Oriental Honey Buzzard | *Pernis ptilorhynchus* | Accipitriformes | Accipitridae | P | LC | Carnivore | II |
| Black Baza | *Aviceda leuphotes* | Accipitriformes | Accipitridae | S | LC | Carnivore | II |
| Crested Serpent Eagle | *Spilornis cheela* | Accipitriformes | Accipitridae | R | LC | Carnivore | II |
| Black Eagle | *Ictinaetus malaiensis* | Accipitriformes | Accipitridae | R | LC | Carnivore | II |
| Crested Goshawk | *Accipiter trivirgatus* | Accipitriformes | Accipitridae | R | LC | Carnivore | II |
| Chinese Sparrowhawk | *Accipiter soloensis* | Accipitriformes | Accipitridae | S | LC | Carnivore | II |
| Japanese Sparrowhawk | *Accipiter gularis* | Accipitriformes | Accipitridae | P | LC | Carnivore | II |
| Besra | *Accipiter virgatus* | Accipitriformes | Accipitridae | R | LC | Carnivore | II |
| Eurasian Sparrowhawk | *Accipiter nisus* | Accipitriformes | Accipitridae | R | LC | Carnivore | II |
| Northern Goshawk | *Accipiter gentilis* | Accipitriformes | Accipitridae | W | LC | Carnivore | II |
| Eastern Marsh Harrier | *Circus spilonotus* | Accipitriformes | Accipitridae | W | LC | Carnivore | II |
| Hen Harrier | *Circus cyaneus* | Accipitriformes | Accipitridae | W | LC | Carnivore | II |
| Pied Harrier | *Circus melanoleucos* | Accipitriformes | Accipitridae | W | LC | Carnivore | II |
| Black Kite | *Milvus migrans* | Accipitriformes | Accipitridae | R | LC | Carnivore | II |
| Grey-faced Buzzard | *Butastur indicus* | Accipitriformes | Accipitridae | P | LC | Carnivore |  |
| Eastern Buzzard | *Buteo japonicus* | Accipitriformes | Accipitridae | W | LC | Carnivore |  |
| Oriental Scops Owl | *Otus sunia* | Strigiformes | Strigidae | R | LC | Carnivore | II |
| Asian Barred Owlet | *Glaucidium cuculoides* | Strigiformes | Strigidae | R | LC | Carnivore | II |
| Little Owl | *Athene noctua* | Strigiformes | Strigidae | R | LC | Carnivore | II |
| Long-eared Owl | *Asio otus* | Strigiformes | Strigidae | W | LC | Carnivore | II |
| Short-eared Owl | *Asio flammeus* | Strigiformes | Strigidae | W | LC | Carnivore | II |
| Common Hoopoe | *Upupa epops* | Bucerotiformes | Upupidae | R | LC | Carnivore |  |
| Dollarbird | *Eurystomus orientalis* | Coraciiformes | Coraciidae | S | LC | Carnivore |  |
| White-throated Kingfisher | *Halcyon smyrnensis* | Coraciiformes | Alcedinidae | R | LC | Carnivore | II |
| Black-capped Kingfisher | *Halcyon pileata* | Coraciiformes | Alcedinidae | S | LC | Carnivore |  |
| Common Kingfisher | *Alcedo atthis* | Coraciiformes | Alcedinidae | R | LC | Carnivore |  |
| Crested Kingfisher | *Megaceryle lugubris* | Coraciiformes | Alcedinidae | R | LC | Carnivore |  |
| Pied Kingfisher | *Ceryle rudis* | Coraciiformes | Alcedinidae | R | LC | Carnivore |  |
| Eurasian Wryneck | *Jynx torquilla* | Piciformes | Picidae | W | LC | Carnivore |  |
| Speckled Piculet | *Picumnus innominatus* | Piciformes | Picidae | R | LC | Carnivore |  |
| Rufous-bellied Woodpecker | *Dendrocopos hyperythrus* | Piciformes | Picidae | P | LC | Carnivore |  |
| Grey-capped Woodpecker | *Dendrocopos canicapillus* | Piciformes | Picidae | R | LC | Carnivore |  |
| Great Spotted Woodpecker | *Dendrocopos major* | Piciformes | Picidae | R | LC | Carnivore |  |
| Grey-headed Woodpecker | *Picus canus* | Piciformes | Picidae | R | LC | Carnivore |  |
| Common Kestrel | *Falco tinnunculus* | Falconiformes | Falconidae | R | LC | Carnivore | II |
| Amur Falcon | *Falco amurensis* | Falconiformes | Falconidae | P | LC | Carnivore | II |
| Merlin | *Falco columbarius* | Falconiformes | Falconidae | W | LC | Carnivore | II |
| Eurasian Hobby | *Falco subbuteo* | Falconiformes | Falconidae | P | LC | Carnivore | II |
| Peregrine Falcon | *Falco peregrinus* | Falconiformes | Falconidae | W | LC | Carnivore | II |
| Black-naped Oriole | *Oriolus chinensis* | Passeriformes | Oriolidae | S | LC | Omnivore |  |
| Black-winged Cuckoo-shrike | *Lalage melaschistos* | Passeriformes | Campephagidae | S | LC | Omnivore |  |
| Swinhoe's Minivet | *Pericrocotus cantonensis* | Passeriformes | Campephagidae | S | LC | Omnivore |  |
| Ashy Minivet | *Pericrocotus divaricatus* | Passeriformes | Campephagidae | P | LC | Carnivore |  |
| Black Drongo | *Dicrurus macrocercus* | Passeriformes | Dicruridae | S | LC | Carnivore |  |
| Ashy Drongo | *Dicrurus leucophaeus* | Passeriformes | Dicruridae | S | LC | Carnivore |  |
| Hair-crested Drongo | *Dicrurus hottentottus* | Passeriformes | Dicruridae | S | LC | Carnivore |  |
| Japanese Paradise-Flycatcher | *Terpsiphone atrocaudata* | Passeriformes | Monarchidae | P | NT | Carnivore |  |
| Tiger Shrike | *Lanius tigrinus* | Passeriformes | Laniidae | S | LC | Carnivore |  |
| Bull-headed Shrike | *Lanius bucephalus* | Passeriformes | Laniidae | P | LC | Carnivore |  |
| Brown Shrike | *Lanius cristatus* | Passeriformes | Laniidae | P | LC | Carnivore |  |
| Long-tailed Shrike | *Lanius schach* | Passeriformes | Laniidae | R | LC | Carnivore |  |
| Chinese Gray Shrike | *Lanius sphenocercus* | Passeriformes | Laniidae | W | LC | Carnivore |  |
| Eurasian Jay | *Garrulus glandarius* | Passeriformes | Corvidae | R | LC | Omnivore |  |
| Azure-winged Magpie | *Cyanopica cyanus* | Passeriformes | Corvidae | R | LC | Omnivore |  |
| Red-billed Blue Magpie | *Urocissa erythroryncha* | Passeriformes | Corvidae | R | LC | Omnivore |  |
| Grey Treepie | *Dendrocitta formosae* | Passeriformes | Corvidae | R | LC | Omnivore |  |
| Common Magpie | *Pica pica* | Passeriformes | Corvidae | R | LC | Omnivore |  |
| Collared Crow | *Corvus pectoralis* | Passeriformes | Corvidae | R | VU | Omnivore |  |
| Large-billed Crow | *Corvus macrorhynchos* | Passeriformes | Corvidae | R | LC | Omnivore |  |
| Coal Tit | *Periparus ater* | Passeriformes | Paridae | R | LC | Carnivore |  |
| Yellow-bellied Tit | *Pardaliparus venustulus* | Passeriformes | Paridae | R | LC | Omnivore |  |
| Cinereous Tit | *Parus cinereus* | Passeriformes | Paridae | R | LC | Carnivore |  |
| Chinese Penduline Tit | *Remiz consobrinus* | Passeriformes | Remizidea | W | LC | Omnivore |  |
| Eurasian Skylark | *Alauda arvensis* | Passeriformes | Alaudidae | W | LC | Omnivore | II |
| Oriental Skylark | *Alauda gulgula* | Passeriformes | Alaudidae | R | LC | Omnivore |  |
| Zitting Cisticola | *Cisticola juncidis* | Passeriformes | Cisticolidae | R | LC | Omnivore |  |
| Plain Prinia | *Prinia inornata* | Passeriformes | Cisticolidae | R | LC | Carnivore |  |
| Oriental Reed Warbler | *Acrocephalus orientalis* | Passeriformes | Acrocephalidae | S | LC | Carnivore |  |
| Black-browed Reed Warbler | *Acrocephalus bistrigiceps* | Passeriformes | Acrocephalidae | S | LC | Carnivore |  |
| Sand Martin | *Riparia riparia* | Passeriformes | Hirundinidae | P | LC | Carnivore |  |
| Barn Swallow | *Hirundo rustica* | Passeriformes | Hirundinidae | S | LC | Carnivore |  |
| Asian House Martin | *Delichon dasypus* | Passeriformes | Hirundinidae | P | LC | Carnivore |  |
| Red-rumped Swallow | *Cecropis daurica* | Passeriformes | Hirundinidae | S | LC | Carnivore |  |
| Collared Finchbill | *Spizixos semitorques* | Passeriformes | Pycnonotidae | R | LC | Omnivore |  |
| Brown-breasted Bulbul | *Pycnonotus xanthorrhous* | Passeriformes | Pycnonotidae | R | LC | Omnivore |  |
| Light-vented Bulbul | *Pycnonotus sinensis* | Passeriformes | Pycnonotidae | R | LC | Omnivore |  |
| Chestnut Bulbul | *Hemixos castanonotus* | Passeriformes | Pycnonotidae | W | LC | Omnivore |  |
| Black Bulbul | *Hypsipetes leucocephalus* | Passeriformes | Pycnonotidae | S | LC | Omnivore |  |
| Dusky Warbler | *Phylloscopus fuscatus* | Passeriformes | Phylloscopidae | W | LC | Carnivore |  |
| Radde's Warbler | *Phylloscopus schwarzi* | Passeriformes | Phylloscopidae | P | LC | Carnivore |  |
| Pallas's Leaf Warbler | *Phylloscopus proregulus* | Passeriformes | Phylloscopidae | W | LC | Carnivore |  |
| Yellow-browed Warbler | *Phylloscopus inornatus* | Passeriformes | Phylloscopidae | W | LC | Carnivore |  |
| Arctic Warbler | *Phylloscopus borealis* | Passeriformes | Phylloscopidae | P | LC | Carnivore |  |
| Two-barred Warbler | *Phylloscopus plumbeitarsus* | Passeriformes | Phylloscopidae | P | LC | Carnivore |  |
| Pale-legged Leaf Warbler | *Phylloscopus tenellipes* | Passeriformes | Phylloscopidae | P | LC | Carnivore |  |
| Eastern Crowned Warbler | *Phylloscopus coronatus* | Passeriformes | Phylloscopidae | P | LC | Carnivore |  |
| Rufous-faced Warbler | *Abroscopus albogularis* | Passeriformes | Cettiidae | S | LC | Carnivore |  |
| Japanese Bush Warbler | *Horornis diphone* | Passeriformes | Cettiidae | W | LC | Carnivore |  |
| Manchurian Bush Warbler | *Horornis canturians* | Passeriformes | Cettiidae | S | LC | Carnivore |  |
| Brownish-flanked Bush Warbler | *Horornis fortipes* | Passeriformes | Cettiidae | R | LC | Carnivore |  |
| Asian Stubtail | *Urosphena squameiceps* | Passeriformes | Cettiidae | P | LC | Carnivore |  |
| Silver-throated Bushtit | *Aegithalos glaucogularis* | Passeriformes | Aegithalidae | R | LC | Carnivore |  |
| Black-throated Bushtit | *Aegithalos concinnus* | Passeriformes | Aegithalidae | R | LC | Carnivore |  |
| Vinous-throated Parrotbill | *Sinosuthora webbiana* | Passeriformes | Sylviidae | R | LC | Omnivore |  |
| Reed Parrotbill | *Paradoxornis heudei* | Passeriformes | Sylviidae | R | NT | Carnivore | II |
| Japanese White-eye | *Zosterops japonicus* | Passeriformes | Zosteropidae | S | LC | Omnivore |  |
| Streak-breasted Scimitar Babbler | *Pomatorhinus ruficollis* | Passeriformes | Timaliidae | R | LC | Omnivore |  |
| Hwamei | *Garrulax canorus* | Passeriformes | Leiothrichidae | R | LC | Omnivore | II |
| Masked Laughingthrush | *Garrulax perspicillatus* | Passeriformes | Leiothrichidae | R | LC | Omnivore |  |
| Lesser Necklaced Laughingthrush | *Garrulax monileger* | Passeriformes | Leiothrichidae | R | LC | Omnivore |  |
| Greater Necklaced Laughingthrush | *Garrulax pectoralis* | Passeriformes | Leiothrichidae | R | LC | Omnivore |  |
| Red-billed Leiothrix | *Leiothrix lutea* | Passeriformes | Leiothrichidae | R | LC | Omnivore | II |
| Eurasian Nuthatch | *Sitta europaea* | Passeriformes | Sittidae | R | LC | Carnivore |  |
| Crested Myna | *Acridotheres cristatellus* | Passeriformes | Sturnidae | R | LC | Omnivore |  |
| Silky Starling | *Spodiopsar sericeus* | Passeriformes | Sturnidae | S | LC | Omnivore |  |
| White-cheeked Starling | *Spodiopsar cineraceus* | Passeriformes | Sturnidae | W | LC | Omnivore |  |
| Black-collared Starling | *Gracupica nigricollis* | Passeriformes | Sturnidae | R | LC | Omnivore |  |
| Common Starling | *Sturnus vulgaris* | Passeriformes | Sturnidae | W | LC | Omnivore |  |
| Siberian Thrush | *Geokichla sibirica* | Passeriformes | Turdidae | P | LC | Omnivore |  |
| White's Thrush | *Zoothera aurea* | Passeriformes | Turdidae | W | LC | Omnivore |  |
| Grey-backed Thrush | *Turdus hortulorum* | Passeriformes | Turdidae | W | LC | Omnivore |  |
| Japanese Thrush | *Turdus cardis* | Passeriformes | Turdidae | S | LC | Omnivore |  |
| Chinese Blackbird | *Turdus mandarinus* | Passeriformes | Turdidae | R | LC | Omnivore |  |
| Eyebrowed Thrush | *Turdus obscurus* | Passeriformes | Turdidae | P | LC | Omnivore |  |
| Pale Thrush | *Turdus pallidus* | Passeriformes | Turdidae | W | LC | Carnivore |  |
| Naumann's Thrush | *Turdus naumanni* | Passeriformes | Turdidae | W | LC | Omnivore |  |
| Dusky Thrush | *Turdus eunomus* | Passeriformes | Turdidae | P | LC | Omnivore |  |
| Rufous-tailed Robin | *Larvivora sibilans* | Passeriformes | Muscicapidae | P | LC | Carnivore |  |
| Siberian Blue Robin | *Larvivora cyane* | Passeriformes | Muscicapidae | P | LC | Carnivore |  |
| Siberian Rubythroat | *Calliope calliope* | Passeriformes | Muscicapidae | P | LC | Carnivore | II |
| Bluethroat | *Luscinia svecica* | Passeriformes | Muscicapidae | P | LC | Carnivore | II |
| Orange-flanked Bluetail | *Tarsiger cyanurus* | Passeriformes | Muscicapidae | P | LC | Omnivore |  |
| Oriental Magpie Robin | *Copsychus saularis* | Passeriformes | Muscicapidae | R | LC | Carnivore |  |
| Daurian Redstart | *Phoenicurus auroreus* | Passeriformes | Muscicapidae | W | LC | Carnivore |  |
| Blue Whistling Thrush | *Myophonus caeruleus* | Passeriformes | Muscicapidae | R | LC | Carnivore |  |
| White-crowned Forktail | *Enicurus leschenaulti* | Passeriformes | Muscicapidae | R | LC | Carnivore |  |
| Siberian Stonechat | *Saxicola maurus* | Passeriformes | Muscicapidae | P | NR | Carnivore |  |
| Blue Rock Thrush | *Monticola solitarius* | Passeriformes | Muscicapidae | R | LC | Carnivore |  |
| White-throated Rock Thrush | *Monticola gularis* | Passeriformes | Muscicapidae | P | LC | Carnivore |  |
| Grey-streaked Flycatcher | *Muscicapa griseisticta* | Passeriformes | Muscicapidae | P | LC | Carnivore |  |
| Dark-sided Flycatcher | *Muscicapa sibirica* | Passeriformes | Muscicapidae | P | LC | Carnivore |  |
| Asian Brown Flycatcher | *Muscicapa dauurica* | Passeriformes | Muscicapidae | P | LC | Carnivore |  |
| Yellow-rumped Flycatcher | *Ficedula zanthopygia* | Passeriformes | Muscicapidae | S | LC | Carnivore |  |
| Narcissus Flycatcher | *Ficedula narcissina* | Passeriformes | Muscicapidae | P | LC | Carnivore |  |
| Mugimaki Flycatcher | *Ficedula mugimaki* | Passeriformes | Muscicapidae | P | LC | Carnivore |  |
| Taiga Flycatcher | *Ficedula albicilla* | Passeriformes | Muscicapidae | P | LC | Carnivore |  |
| Blue-and-white Flycatcher | *Cyanoptila cyanomelana* | Passeriformes | Muscicapidae | P | LC | Carnivore |  |
| Goldcrest | *Regulus regulus* | Passeriformes | Regulidae | W | LC | Carnivore |  |
| Japanese Waxwing | *Bombycilla japonica* | Passeriformes | Bombycillidae | W | NT | Omnivore |  |
| White-rumped Munia | *Lonchura striata* | Passeriformes | Estrildidae | R | LC | Omnivore |  |
| Scaly-breasted Munia | *Lonchura punctulata* | Passeriformes | Estrildidae | R | LC | Omnivore |  |
| Russet Sparrow | *Passer cinnamomeus* | Passeriformes | Passeridae | S | LC | Omnivore |  |
| Eurasian Tree Sparrow | *Passer montanus* | Passeriformes | Passeridae | R | LC | Omnivore |  |
| Forest Wagtail | *Dendronanthus indicus* | Passeriformes | Motacillidae | S | LC | Carnivore |  |
| Eastern Yellow Wagtail | *Motacilla tschutschensis* | Passeriformes | Motacillidae | P | LC | Carnivore |  |
| Gray Wagtail | *Motacilla cinerea* | Passeriformes | Motacillidae | P | LC | Carnivore |  |
| White Wagtail | *Motacilla alba* | Passeriformes | Motacillidae | R | LC | Carnivore |  |
| Richard's Pipit | *Anthus richardi* | Passeriformes | Motacillidae | S | LC | Carnivore |  |
| Olive-backed Pipit | *Anthus hodgsoni* | Passeriformes | Motacillidae | W | LC | Carnivore |  |
| Pechora Pipit | *Anthus gustavi* | Passeriformes | Motacillidae | P | LC | Carnivore |  |
| Red-throated Pipit | *Anthus cervinus* | Passeriformes | Motacillidae | W | LC | Carnivore |  |
| Buff-bellied Pipit | *Anthus rubescens* | Passeriformes | Motacillidae | W | LC | Carnivore |  |
| Water Pipit | *Anthus spinoletta* | Passeriformes | Motacillidae | W | LC | Carnivore |  |
| Brambling | *Fringilla montifringilla* | Passeriformes | Fringillidae | W | LC | Omnivore |  |
| Hawfinch | *Coccothraustes coccothraustes* | Passeriformes | Fringillidae | W | LC | Omnivore |  |
| Chinese Grosbeak | *Eophona migratoria* | Passeriformes | Fringillidae | S | LC | Omnivore |  |
| Japanese Grosbeak | *Eophona personata* | Passeriformes | Fringillidae | P | LC | Omnivore |  |
| Common Rosefinch | *Carpodacus erythrinus* | Passeriformes | Fringillidae | P | LC | Herbivore |  |
| Grey-capped Greenfinch | *Chloris sinica* | Passeriformes | Fringillidae | R | LC | Herbivore |  |
| Eurasian Siskin | *Spinus spinus* | Passeriformes | Fringillidae | W | LC | Omnivore |  |
| Meadow Bunting | *Emberiza cioides* | Passeriformes | Emberizidae | R | LC | Omnivore |  |
| Tristram's Bunting | *Emberiza tristrami* | Passeriformes | Emberizidae | P | LC | Omnivore |  |
| Chestnut-eared Bunting | *Emberiza fucata* | Passeriformes | Emberizidae | W | LC | Omnivore |  |
| Little Bunting | *Emberiza pusilla* | Passeriformes | Emberizidae | W | LC | Omnivore |  |
| Yellow-browed Bunting | *Emberiza chrysophrys* | Passeriformes | Emberizidae | P | LC | Omnivore |  |
| Rustic Bunting | *Emberiza rustica* | Passeriformes | Emberizidae | W | VU | Omnivore |  |
| Yellow-throated Bunting | *Emberiza elegans* | Passeriformes | Emberizidae | P | LC | Omnivore |  |
| Chestnut Bunting | *Emberiza rutila* | Passeriformes | Emberizidae | P | LC | Omnivore |  |
| Black-faced Bunting | *Emberiza spodocephala* | Passeriformes | Emberizidae | W | LC | Omnivore |  |
| Pallas's Bunting | *Emberiza pallasi* | Passeriformes | Emberizidae | W | LC | Omnivore |  |
| Ochre-rumped Bunting | *Emberiza yessoensis* | Passeriformes | Emberizidae | W | NT | Omnivore |  |
| Reed Bunting | *Emberiza schoeniclus* | Passeriformes | Emberizidae | W | LC | Omnivore |  |
